# Netrin‐1 Inhibits Neuroinflammation by Modulating DRD2/GSK3β Signaling and Suppressing ROS in a Parkinson's Disease Model

**DOI:** 10.1111/cns.70651

**Published:** 2025-11-17

**Authors:** Lee Ya Kim, Seung Hoon Jeon, Yumin Heo, Eun Ji Kang, Dae Ki Hong, Seong Su Kang, Minwoo Lee, Eun Hee Ahn

**Affiliations:** ^1^ Department of Physiology, College of Medicine Hallym University Chuncheon‐si Gangwon‐Do South Korea; ^2^ Department of Pathology and Laboratory Medicine Emory University School of Medicine Atlanta Georgia USA; ^3^ Department of Neurology Hallym University Sacred Heart Hospital Anyang Republic of Korea; ^4^ Department of Neurology, College of Medicine Hallym University Chuncheon‐si Gangwon‐Do South Korea; ^5^ Institute of Medical Science, College of Medicine Hallym University Chuncheon Republic of Korea

**Keywords:** anti‐inflammatory effects, DRD2/GSK3β signaling, Netrin‐1, Parkinson's disease, ROS, therapeutic protein molecule

## Abstract

**Background:**

Netrin‐1 is stably expressed in mature neurons, where it regulates synaptic plasticity, promotes neuronal survival, and modulates cell adhesion and migration. However, the molecular link between Netrin‐1 and the pathogenesis of Parkinson's disease (PD) has not yet been clearly elucidated.

**Aims:**

In this study, we investigated the neuroprotective effects of Netrin‐1 against dopaminergic neuronal death associated with PD pathology.

**Results:**

Here, we show that in a rotenone‐induced cellular model, Netrin‐1 treatment significantly reduced reactive oxygen species (ROS) production, α‐synuclein phosphorylation, and subsequent apoptosis. Moreover, Netrin‐1 suppressed the expression of pro‐inflammatory cytokines, including IL‐6, TNF‐α, and IL‐1β, and promoted the activation of dopamine D2 receptor (DRD2) signaling, which is crucial for dopaminergic neuron survival. Of note, in the Netrin‐1 conditional knockout mouse model, we observed significant dopaminergic neuronal loss, accompanied by increased α‐synuclein hyperphosphorylation at Ser129 and elevated levels of the truncated form of deleted in colorectal cancer (DCC) generated by activated caspase‐3, a marked depletion of DRD2 expression, and hyperphosphorylation of GSK3β at Tyr216. In contrast, these pathological features were attenuated in Netrin‐1 wild‐type (WT) mice. Additionally, the aging process in α‐synuclein (SNCA) transgenic (Tg) mice was characterized by reduced levels of Netrin‐1 and DRD2, alongside increased α‐synuclein accumulation, proinflammatory cytokines, and caspase‐3 activation.

**Conclusion:**

These findings suggest that Netrin‐1 protects dopaminergic neurons by regulating neuroinflammation, preserving DRD2 signaling, and inhibiting phosphorylation of GSK3β at Tyr216, thereby offering potential as a therapeutic agent for dopaminergic neurodegeneration.

## Graphical abstract

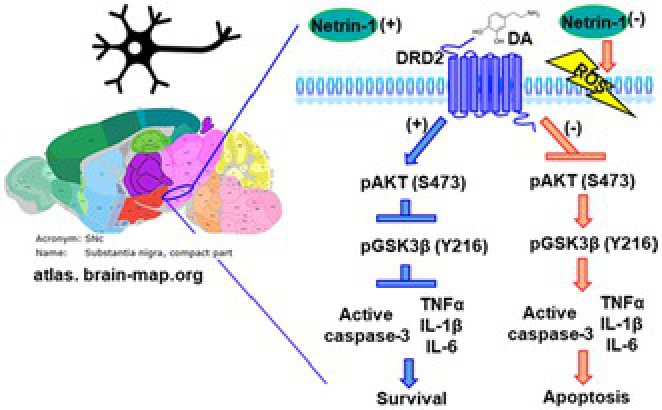

**Abbreviations**
AKT: protein kinase B
CC‐3: cleaved caspase‐3
DA: dopamine
DCC: deleted in colorectal cancer
DRD2: dopamine receptor D2
GSK3β: glycogen synthase kinase 3 beta
Iba1: ionized calcium binding adaptor molecule 1
IL‐1β: interleukin‐1 beta
IL‐6: interleukin 6
NTN1: Netrin‐1
rNetrin‐1: recombinant Netrin‐1
SN: substantia nigra
SNCA: α‐synuclein
TH: tyrosine hydroxylase
TNF‐α: tumor necrosis factor‐alpha
VTA: ventral tegmental area

## 1 Introduction

Netrin‐1, a secreted laminin‐related protein, is well known for its role in axon guidance and neuronal migration during embryonic development [1]. In the adult central nervous system, Netrin‐1 remains expressed in post‐mitotic neurons, where it contributes to synaptic plasticity, cell adhesion, and neuronal survival [2]. Its receptor, Deleted in Colorectal Cancer (DCC), functions not only as an axon guidance cue receptor but also as a dependence receptor that promotes cell survival in the presence of Netrin‐1 and triggers apoptosis when unbound [3]. Notably, DCC is enriched in midbrain dopaminergic neurons [4, 5], and recent studies suggest that Netrin‐1/DCC signaling may support neuronal survival through activation of pathways such as PI3K/Akt and MAPK/ERK, although direct evidence in dopaminergic neurons remains to be fully elucidated [6, 7, 8].

Also, dopamine is a critical neurotransmitter in the central nervous system, exerting its effects through five distinct receptor subtypes (D1–D5). These receptors are categorized into two families based on their structural and functional characteristics: the D1‐like receptors (D1 and D5), which typically stimulate adenylyl cyclase activity, and the D2‐like receptors (D2, D3, and D4), which generally inhibit this enzyme. Each receptor subtype exhibits unique expression patterns and plays specific roles in modulating motor control, cognition, and emotional responses [9]. Among the dopamine receptors, D2 receptors are abundantly expressed in the substantia nigra pars compacta (SNpc) of both humans and rodents, and their degeneration is a hallmark of Parkinson's disease (PD). They function as autoreceptors to regulate dopamine synthesis and release, thereby maintaining dopaminergic neuron homeostasis [10]. And, D1 receptors are predominantly found in the striatum [11], particularly within medium spiny neurons (MSNs), which are integral to the direct pathway of the basal ganglia circuitry [12]. This differential distribution underscores the distinct yet complementary roles of D1 and D2 receptors in modulating dopaminergic signaling and neuronal survival.

One such pathway involves glycogen synthase kinase‐3 beta (GSK3β), a serine/threonine kinase implicated in neuronal apoptosis. Activation of DRD2 has been associated with the inhibition of GSK3β activity, thereby promoting neuronal survival. Conversely, overactivation of GSK3β contributes to dopaminergic neuron death, and its inhibition has been shown to protect these neurons in PD models [13]. Despite these insights, the interplay between Netrin‐1/DCC signaling and DRD2‐mediated GSK3β modulation in the context of dopaminergic neuron survival remains. Understanding this relationship could provide novel insights into the mechanisms underlying dopaminergic neuron degeneration in PD and inform the development of targeted therapeutic strategies. Therefore, we investigated the neuroprotective role of Netrin‐1 in dopaminergic neurons, focusing on its regulation of DRD2/GSK3β signaling and its potential to suppress ROS generation key pathways implicated in Parkinson's disease progression.

## 2 Materials and Methods

### 2.1 Mice and Cell Lines

SNCA‐null mice (B6;129X1‐Sncatm1Rosl, stock# 003692) and human SNCA‐overexpressing mice (B6.Cg Tg(SNCA)OVX37RwmSncatm1Rosl/J, stock# 023837) on a pure genetic background were back‐crossed. SH‐SY5Y cells were cultured in DMEM/F12 added with 10% FBS and penicillin (100 units/mL)–streptomycin (100 μg/mL) (all from Hyclone). Cells were incubated at 37°C in a humidified atmosphere of 5% CO_2_.

### 2.2 TUNEL Assay

Apoptosis in SH‐SY5Y cells and dopaminergic neurons was detected using the In Situ Cell Death Detection Kit, TMR Red (Abcam, Cat# Ab66110). The apoptotic index was calculated as the percentage of TUNEL‐positive cells among the total number of TH‐positive cells.

### 2.3 Gene‐Expression Profiling Dataset

DRD1, DRD2, DRD3, DRD4, and DRD5 gene expression profiling in the substantia nigra (SN) of PD patients was conducted using a gene dataset available on the GEO repository (GDS2821 accession number). Gene expression profiling was done using Affymetrix Human Genome U133 Plus 2.0 GeneChip arrays. The STRING database was used to investigate actual molecular interactions and common pathways among NTN1, SNCA, DRD2, and GSK3β. Data were collected from primary studies found in various databases, including InterPro, Reactome and BioGRID.

### 2.4 Measurement of Reactive Oxygen Species (ROS) Formation

The DCFH‐DA method was used to detect the levels of intracellular reactive oxygen species (ROS). After indicated treatments, the cells were collected and then incubated with 5 μM DCFH‐DA (see Table S2). After incubation for 2 h at 37°C, fluorescence images were taken with a fluorescence microscope at an excitation/emission of 520/605 nm (Lionheart, BioTek USA).

### 2.5 Animals

All animals were housed in filter‐topped cages under a 12 h light/dark cycle at an ambient temperature of 22°C. Females and males were kept separately. Tap water and rodent chow were available ad libitum. Investigators were blinded to the group allocation during the animal experiments.

### 2.6 B6.129(SJL)‐Ntn1^tm1.1Tek^/J Mice Conditional KO Mice

Three‐month‐old B6.129(SJL)‐Ntn1^tm1.1Tek^/J mice (C57BL/6 background) were obtained from the Jackson Laboratory (Stock no: 028038). These mutant mice possess loxP sites flanking the first exon of the *Ntn1* gene. Homozygous mice are viable and fertile. Removal of the floxed sequence by cre recombinase generates a null allele resulting in the silencing of Netrin‐1 in the cre‐targeted tissue. The generation of the Netrin‐1^*fl*^ allele and Netrin‐1^*fl*^ mice is described in and available on the Jackson Laboratory website. Netrin‐1 knockout in the SN was achieved by injecting stereotaxically AAV6‐Cre virus (vs AAV6‐Control virus) into the right SN of three‐month‐old B6.129(SJL)‐Ntn1^tm1.1Tek^/J mice. Mice were randomly allocated to experimental groups. Animal care and handling were performed according to the Emory Medical School guidelines. The protocol was reviewed and approved by the Emory Institutional Animal Care and Use Committee.

### 2.7 Stereotaxic Injection and Treatments

Stereotaxic coordinates were determined according to the rat brain atlas of Paxinos and Watson (1997) and the mouse brain atlas of Paxinos and Franklin (2001). *AAV6 virus injections in* three‐month‐old B6.129(SJL)‐Ntn1^tm1.1Tek^/J mice. The AAV6‐Cre virus (with Capsid from AAV6 and ITR from AAV2) was used to KO *Netrin‐1* expression in B6.129(SJL)‐Ntn1^tm1.1Tek^/J mice. This AAV serotype 6 virus expresses Cre Recombinase (tagged with nuclear localization sequence) and, was driven by CMV promoter. AAV6‐null virus (with Capsid from AAV6 and ITR from AAV2) expressing non‐functional vector under the control of the promoter CMV was used as a control virus. AAV6‐Cre and AAV6‐null viruses were purchased from Vector Biolabs (Cat. No: 7013 and 7029, respectively). Three‐month‐old B6.129(SJL)‐Ntn1^tm1.1Tek^/J mice were anesthetized with isoflurane. Meloxicam (5 mg/kg) was injected subcutaneously for analgesia (Loxicom, Norbrook, USA). Unilateral stereotaxic injection of AAV6‐Cre or control AAV6‐null viruses was performed at coordinates corresponding to the substantia nigra: anteroposterior (A/P): −3.1 mm, mediolateral (M/L): ±1.2 mm from Bregma, and dorsoventral (D/V): 4.3 mm from the dura surface. Each site received 2 μL of viral constructions at a rate of 0.25 μL/min, using a 10 μL Hamilton syringe. Viral titer was 1 × 10^12^ vg/mL for AAV6 virus constructions. The needle was kept in place for 5 min after the injection was completed, then gently removed. Mice were placed on a heating pad until they recovered from the anesthesia.

### 2.8 Behavioral Tests

Three‐month‐old B6.129(SJL)‐Ntn1^tm1.1Tek^/J mice loss of motor function was tested 8 weeks following AAV6‐Cre virus injection. Footprint test: The dye was applied to the soles of the hind paws, and the mice were allowed to walk on a narrow white paper‐covered corridor [14]. At least 45 sequential steps were used to determine the mean values for each measurement, including stride angle, stride length, foot length, step width, toe spread, and paw area [15]. The measurement was made using ImageJ software. Cylinder test: Mice were placed individually into a glass cylinder (12 cm diameter × 22 cm height) and were recorded with a video camera. The recorded files were analyzed by a blinded observer. Between 20 and 30 wall touches per animal (contacts with fully extended digits executed with the forelimb ipsilateral and contralateral to the lesion) were counted. Nest building test: Mice were singly housed with wood‐chip bedding with no environmental enrichment items and had ad libitum access to food and water throughout the experiments. The nestlet was manufactured from cotton fiber, sterilized during manufacture, and cleanly packed. A single piece of nestlet was located in the middle of each mouse cage at 11 a.m. Next morning at 11 a.m., the amounts of torn or untorn nestlet materials in each mouse cage were weighed. Tail suspension test: Tape was adhered to the tails of mice, after the camera was placed in position. Mice were suspended by placing the free end of the tape on the suspension bar. Their behaviors were recorded for 3 min and the immobilized time was analyzed.

### 2.9 Immunohistochemistry

Transcardially perfused mice brains using PBS and 4% paraformaldehyde (PFA) were cut to 30 μm thickness using a cryotome and performed with the HRP/DAB IHC detection kit (ab236466) according to the manufacturer's instructions. Briefly, sections were incubated in 3% hydrogen peroxide for blockage of endogenous peroxidase → tissue blocking (supplied blocking buffer) → primary antibody overnight at 4°C → HRP‐conjugated secondary antibody which reacted with DAB chromogen. After chromogen incubation, counterstaining was applied using Mayer's hematoxylin solution (51,275, Sigma Aldrich). To detect double antigens, the sections were incubated with a mixed Alexa Fluor 488 and 594 conjugated secondary antibody for 2 h at room temperature.

### 2.10 Immunofluorescence

Free‐floating slices were rinsed in PBS then permeabilized and blocked with PBS‐BT (50 mM Tris–HCl, 150 mM NaCl, 3% bovine serum albumin (BSA), 0.1% Triton X‐100, pH 7.4) blocking solution for 1 h. Afterward, the sections were incubated with primary antibodies (see Table S1) in a PBS‐BT solution on a shaker overnight at 4°C. The next day, sections were rinsed and incubated with corresponding secondary antibodies directly conjugated with fluorophores (1:3000 dilution, Alexa Fluor 488, Alexa Fluor 594, and Cy5 conjugate from Abcam) for 2 h at room temperature. Finally, slices were rinsed in PBS and mounted (Invitrogen, F4680).

### 2.11 Immunoblotting

Cells and brain samples were lysed and, if necessary, homogenized in lysis buffer (50 mM Tris, pH 7.4, 40 mM NaCl, 1 mM EDTA, 0.5% Triton X‐100, 1.5 mM Na_3_VO_4_, 50 mM NaF, 10 mM sodium pyrophosphate, and 10 mM sodium β‐glycerophosphate, supplemented with a cocktail of protease inhibitors). Lysates were then centrifuged at 15,000 rpm for 20 min at 4°C and the protein concentration of the supernatant was measured with the Pierce BCA Protein Assay kit (Part no. 23225). The supernatant was denatured at 95°C in Laemmli buffer. After loading and running proteins in an SDS–PAGE gel, the samples were transferred to a nitrocellulose membrane. Membrane blocking and antibody staining were performed according to the primary antibody manufacturer's instructions. Information on the primary antibodies used to analyze specific molecules can be found in Table S1.

### 2.12 Cytokine ELISA

Mouse brain (SN) and SH‐SY5Y samples were lysed in lysis buffer (50 mM Tris, pH 7.4, 40 mM NaCl, 1 mM EDTA, 0.5% Triton X‐100, 1.5 mM Na_3_VO_4_, 50 mM NaF, 10 mM sodium pyrophosphate and 10 mM sodium β‐glycerophosphate, supplemented with a cocktail of protease inhibitors), and centrifuged at 15,000 rpm for 15 min at 4°C for cell lysate or ultracentrifuged for 20 min at 4°C 100,000 rpm for mouse colon and brain tissues. After centrifugation, isolated supernatant part for cytokine (TNF‐α, IL‐6 and IL‐1β) levels analysis used ELISA kits (see Table S3).

### 2.13 Quantification and Statistical Analysis

All data are expressed as mean ± SEM from three or more independent experiments. Representative morphological images obtained from at least 3 experiments with similar results were provided. ImageJ 1.47 software was used to analyze IHC and IF experiments, and Image Lab software for western blot analysis. The statistical analysis of results was performed using GraphPad Prism 5 software. All data were tested for normal distribution in order to analyze results accordingly using parametric or non‐parametric tests. To compare results between two groups, the Student's unpaired *t*‐test was used. When more than two groups were compared, one‐way ANOVA followed by Tukey post hoc test was applied. For repeated measures, a Repeated‐Measures (RM) ANOVA or two‐way ANOVA test was performed followed by Tukey multiple comparisons post hoc test. Assessments with *p* < 0.05 were considered significant.

## 3 Results

### 3.1 DRD2 Is Selectively Expressed in the Substantia Nigra Is Reduced in PD and Correlates With Pathogenic Signaling

**FIGURE 1:**
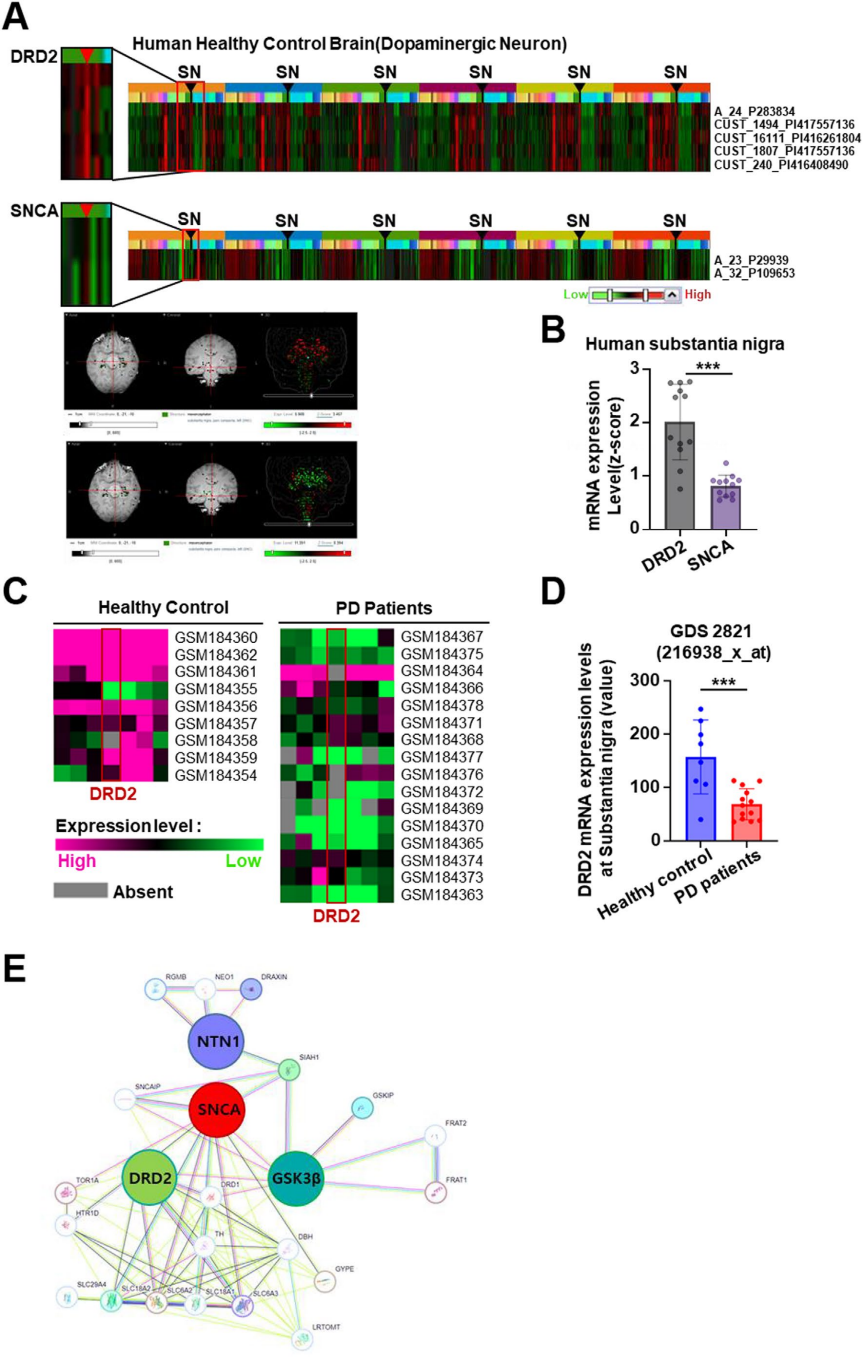
Spatial and comparative gene expression of DRD2 and SNCA in the human substantia nigra and their molecular interactions relevant to Parkinson's disease. (A) Heatmaps showing mRNA expression patterns of DRD2 and SNCA in the substantia nigra (SN) of healthy human brains enriched with dopaminergic neurons. Probe IDs used for the analysis are indicated. Representative brain images depict spatial expression patterns corresponding to the respective probes from the Allen Brain Atlases (https://portal.brain‐map.org). (B) Quantification of mRNA expressions (Z‐Score) of DRD2 and SNCA, in the human substantia nigra (SN). Bars and error bars represent the mean ± SEM. Statistical significance was determined by one‐way ANOVA with Tukey's post hoc test. ***p < 0.001. (C) Heatmap of DRD2 mRNA expression in the substantia nigra of healthy controls and Parkinson's disease (PD) patients, based on GEO dataset samples. Expression levels are color‐coded from high (magenta) to low (green). (D) Comparison of DRD2 mRNA expression in the substantia nigra between healthy controls and PD patients, using GEO dataset GDS2821 (probe ID: 216983_x_at). (E) Predicted protein–protein interaction network including DRD2, SNCA, GSK3β, and NTN1, generated using STRING. Node size reflects interaction confidence, and edge colors represent supporting evidence (e.g., experimental data, curated databases, or co‐expression).

In Parkinson's disease (PD), the degeneration of dopaminergic neurons disrupts dopamine D2 receptor (DRD2) signaling, contributing to both motor impairments and neuropsychiatric symptoms [16, 17]. Clinically, dopamine replacement therapies such as L‐DOPA administration or the use of DRD2‐targeting agonists are commonly employed to restore dopaminergic tone [18, 19]. While prior studies have highlighted a potential interaction between DRD2 and the SNCA gene [20, 21], and the involvement of DRD2 in SNCA‐associated signaling cascades has been proposed, the molecular mechanisms incorporating Netrin‐1 recently identified as a direct SNCA‐binding partner remain poorly characterized [22, 23]. To investigate the relevance of DRD2 mRNA levels in the human substantia nigra, we confirmed strong DRD2 mRNA expression in the healthy substantia nigra brain region. In contrast, SNCA expression was markedly reduced, consistent with its established association with Parkinson's disease progression. These results were obtained from publicly available datasets in the Allen Human Brain Atlas (Figure 1A,B) and the Gene Expression Omnibus (GEO) database GDS2821 set (Figure 1C,D). Of note, in the healthy human substantia nigra, DRD1, DRD3, DRD4, and DRD5 did not exhibit an expression pattern opposite to that of the Parkinson's disease‐associated molecule SNCA (Figure S1A,B). Also, we examined the differential regional expression patterns of dopamine receptors DRD1 and DRD2 in relation to the dopaminergic neuronal circuitry in the mouse brain. Immunostaining analysis showed that DRD2 was predominantly expressed in the substantia nigra, where the soma of dopaminergic neurons is located (Figure S2A). In contrast, DRD1 expression was primarily observed in the striatum, the target region of dopaminergic neuronal projections (Figure S2B). Furthermore, we successfully analyzed the gene network interactions among DRD2, SNCA, NTN1, and GSK3β using the STRING database (Figure 1E).

### 3.2 Rotenone Induced Cell Toxicity via ROS Generation and DRD2 Reduction Accompanied by SNCA Hyperphosphorylation

**FIGURE 2:**
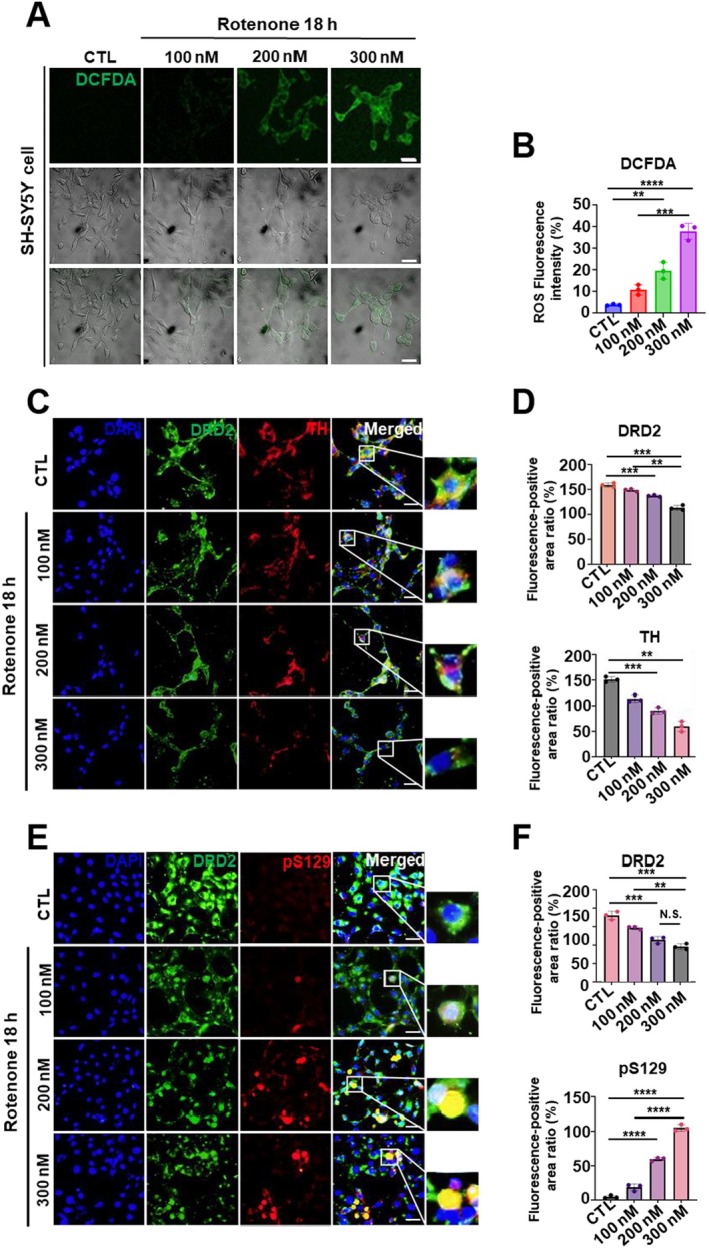
Rotenone‐induced oxidative stress and its modulation of DRD2‐associated pathogenic molecular in SH‐SY5Y cells. (A) Representative images of DCFDA staining in SH‐SY5Y cells treated with increasing concentrations of rotenone (100, 200, and 300 nM) for 18 h, showing enhanced generation of reactive oxygen species (ROS). Top panels show DCFDA fluorescence (green); middle, brightfield images; bottom, merged images. (B) Quantification of DCFDA fluorescence intensity. Data are mean ± SEM. Statistical analysis was performed using one‐way ANOVA followed by Tukey's post hoc test. *N* = 3 per group. (C) Representative immunofluorescence images of SH‐SY5Y cells treated with rotenone (100, 200, 300 nM), showing co‐localization of dopaminer D2 receptor (DRD2, green) and tyrosine hydroxylase (TH, red). Nuclei are counterstained with DAPI (blue). Merged images include magnified views of boxed regions. (D) Quantification bar graphs of fluorescence intensity in DRD2 (upper) and TH (lower) positive areas. (E) Representative immunofluorescence images showing co‐localization of dopamine D2 receptor (DRD2, green) and phosphorylated α‐synuclein at Ser129 (pS129, red). Nuclei were stained with DAPI (blue). Merged images include magnified views. (F) Quantification of fluorescence intensity of DRD2 (upper bar graph) and α‐synuclein pS129 (lower bar graph) in SH‐SY5Y cells. Data are mean ± SEM. Statistical significance was determined by one‐way ANOVA with Tukey's post hoc test. N.S., not significant; **p < 0.01; ***p < 0.001; ****p < 0.0001. Scale bar: 50 μm.

Rotenone, a mitochondrial complex I inhibitor, induces reactive oxygen species (ROS), which in turn trigger α‐synuclein phosphorylation at Ser129, a modification closely associated with Parkinson's disease pathology. To validate that rotenone indeed triggers ROS production, we performed a DCF‐DA assay on SH‐SY5Y cells treated with various concentrations of rotenone (100–300 nM). Rotenone treatment led to a dose‐dependent increase in intracellular ROS levels, as indicated by enhanced green fluorescence observed under a fluorescence microscope (Figure 2A,B). Using co‐immunofluorescence staining, we investigated the expression levels of DRD2 and tyrosine hydroxylase (TH) in response to ROS overproduction. In the SH‐SY5Y cell in vitro model, we observed that elevated ROS levels led to a concomitant reduction in both DRD2 and TH expression (Figure 2C,D). In addition, we observed simultaneous α‐synuclein hyperphosphorylation and DRD2 downregulation, both of which are key pathological features in Parkinson's disease. These observations suggest that DRD2 may be functionally linked to oxidative stress–induced neurotoxicity and cellular damage, thereby contributing to PD pathogenesis (Figure 2E,F).

### 3.3 rNetrin‐1 Prevents Apoptosis via DRD2‐Mediated GSK3β/AKT Signaling and Inhibited Proinflammatory Cytokines in Cellular PD Model

**FIGURE 3:**
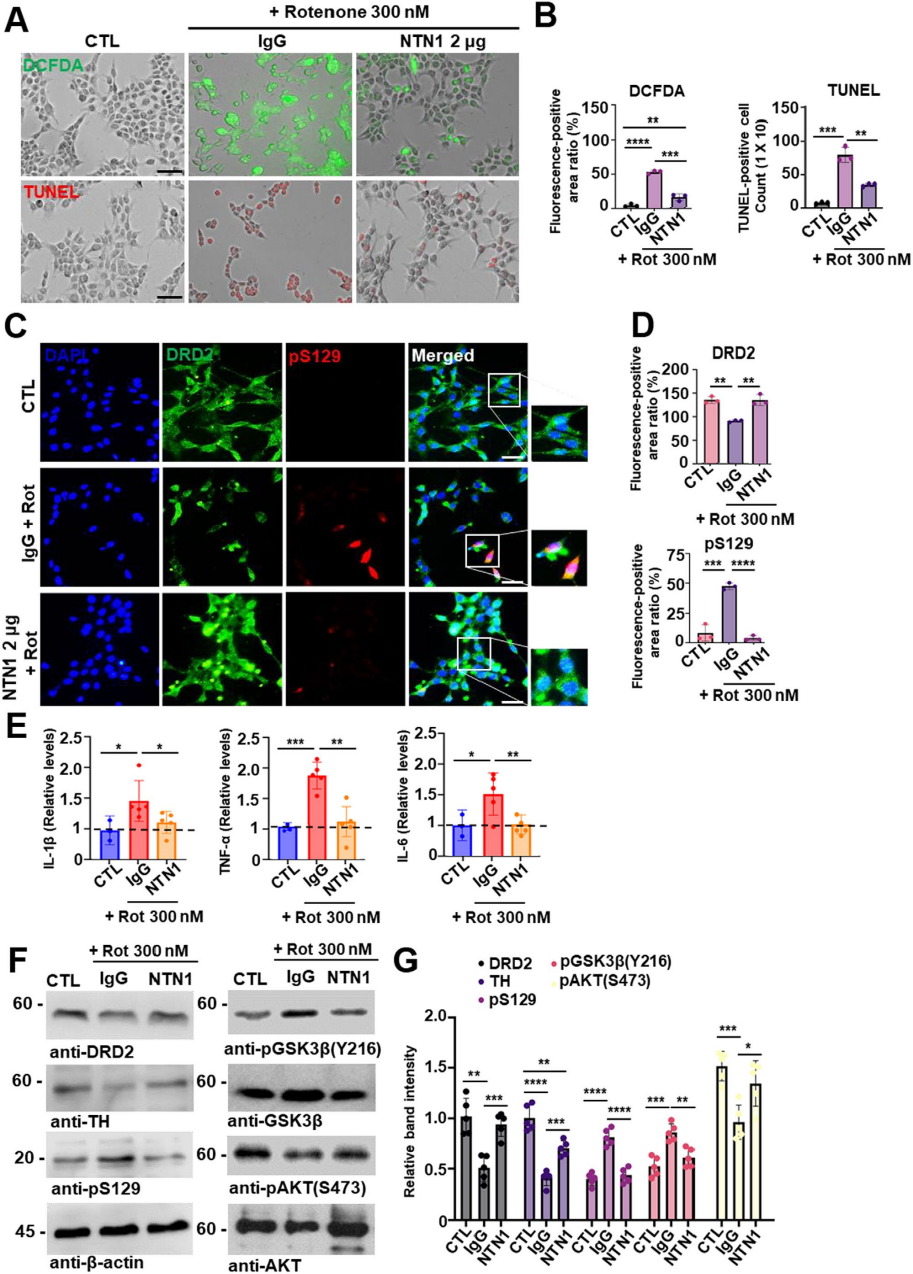
Neuroprotective effects of Netrin‐1 against rotenone‐induced ROS and proinflammatory cytokines in a cellular model. (A) Representative fluorescence images of SH‐SY5Y cells pretreated with control IgG or recombinant Netrin‐1 (rNetrin‐1: 2 μg for 12 h), followed by rotenone treatment (300 nM for 18 h). Intracellular ROS levels were assessed using DCFDA (green), and DNA fragmentation was detected by the TUNEL staining (red), respectively. CTL indicates untreated control group. Scale bar: 100 μm. (B) Quantification of DCFDA green fluorescence intensity (upper bar graph) and the number of TUNEL‐positive cells (lower bar graph). Data are mean ± SEM. Statistical significance was determined by one‐way ANOVA with Tukey's post hoc test. *N* = 3. **p < 0.01; ***p < 0.001; ****p < 0.0001. (C) Representative immunofluorescence images showing co‐localization of DRD2 (green) and pS129 (red) in SH‐SY5Y cells pretreated with rNetrin‐1 or CTL IgG (12 h), followed by rotenone exposure (300 mM for 18 h). Nuclei are counterstained with DAPI (blue). Merged images include magnified views of the indicated regions. CTL indicates the untreated pure control group. Scale bar: 50 μm. (D) Quantification of fluorescence intensity of DRD2 (upper bar graph) and α‐synuclein pS129 (lower bar graph) in SH‐SY5Y cells. Data are mean ± SEM. Statistical analysis was performed using one‐way ANOVA with Tukey's post hoc test. *N* = 3. **p < 0.01; ***p < 0.001; ****p < 0.0001. (E) ELISA analysis of proinflammatory cytokines (IL‐1β, TNF‐α and IL‐1) pretreated with rNetrin‐1 or IgG (12 h), followed by rotenone exposure (300 nM for 18 h). Cytokine levels were normalized to untreated control and presented as relative values. Data are mean ± SEM. Statistical significance was determined using one‐way ANOVA with Tukey's post hoc test. (F) Immunoblot analysis of CTL group, rotenone + IgG group, and rotenone + rNetrin‐1 group. Each in vitro group was analyzed in triplicate for immunoblotting. (G) Western blot band densitometer bar graph. Bars and error bars represent the mean ± SEM. Statistical significance was determined using a one‐way ANOVA followed by post hoc Tukey test for multiple group comparison. CTL *N* = 5; rotenone + IgG *N* = 5; rotenone + rNetrin‐1 *N* = 5 each group; *p < 0.05; **p < 0.01; ***p < 0.001; ****p < 0.0001; N.S., not significant.

Netrin‐1 has been proposed to modulate the transcription factor C/EBPβ, which regulates the expression of key proinflammatory mediators including IL‐6, IL‐1β, TNF‐α, and NF‐κB, thereby implicating Netrin‐1 in the suppression of neuroinflammatory signaling pathways relevant to Parkinson's disease pathogenesis [24, 25, 26, 27, 28]. To address the therapeutic potential of Netrin‐1 as a protein‐based treatment, recombinant Netrin‐1 (rNetrin‐1) was administered to SH‐SY5Y cells 12 h prior to exposure to the neurotoxin rotenone. After that, cells were subsequently treated with 300 nM rotenone for 18 h. In the group pre‐treated with 2 μg of rNetrin‐1, intracellular ROS levels and the number of TUNEL‐positive cells were significantly reduced compared to the IgG‐treated group (Figure 3A,B). Moreover, rNetrin‐1 inhibited α‐synuclein phosphorylation at Ser129, an effect not observed in the IgG + rotenone treated group (Figure 3C,D). We also assessed proinflammatory cytokine levels in rNetrin‐1 + rotenone and IgG + rotenone groups. Proinflammatory cytokines ELISA analysis revealed that IL‐1β, TNF‐α, and IL‐6 levels were significantly reduced in the rNetrin‐1 treated group compared to the control IgG group (Figure 3E). Next, we investigated whether rNetrin‐1 stimulates DRD2 signaling and induces phosphorylation of downstream molecules, including GSK3β and AKT, using immunoblotting analysis. Recent studies have shown that Tyr216 phosphorylation enhances GSK3β kinase activity, which in turn promotes hyperphosphorylation of its downstream targets, contributing to protein misfolding and aggregation [29, 30, 31]. In Parkinson's disease models, GSK3β activation has been shown to facilitate α‐synuclein aggregation and dopaminergic neuronal loss through mechanisms involving mitochondrial dysfunction, oxidative stress, and proinflammatory signaling pathways such as NF‐κB [32, 33, 34]. Notably, Netrin‐1 abolished GSK3β hyperphosphorylation at Tyr216, while increased phosphorylation of AKT at Ser473 was associated with inhibition of α‐synuclein hyperphosphorylation, as confirmed by immunoblotting analysis (Figure 3F,G).

### 3.4 Severe Neuronal Loss in the SN of Netrin‐1 Conditional KO Mice

**FIGURE 4:**
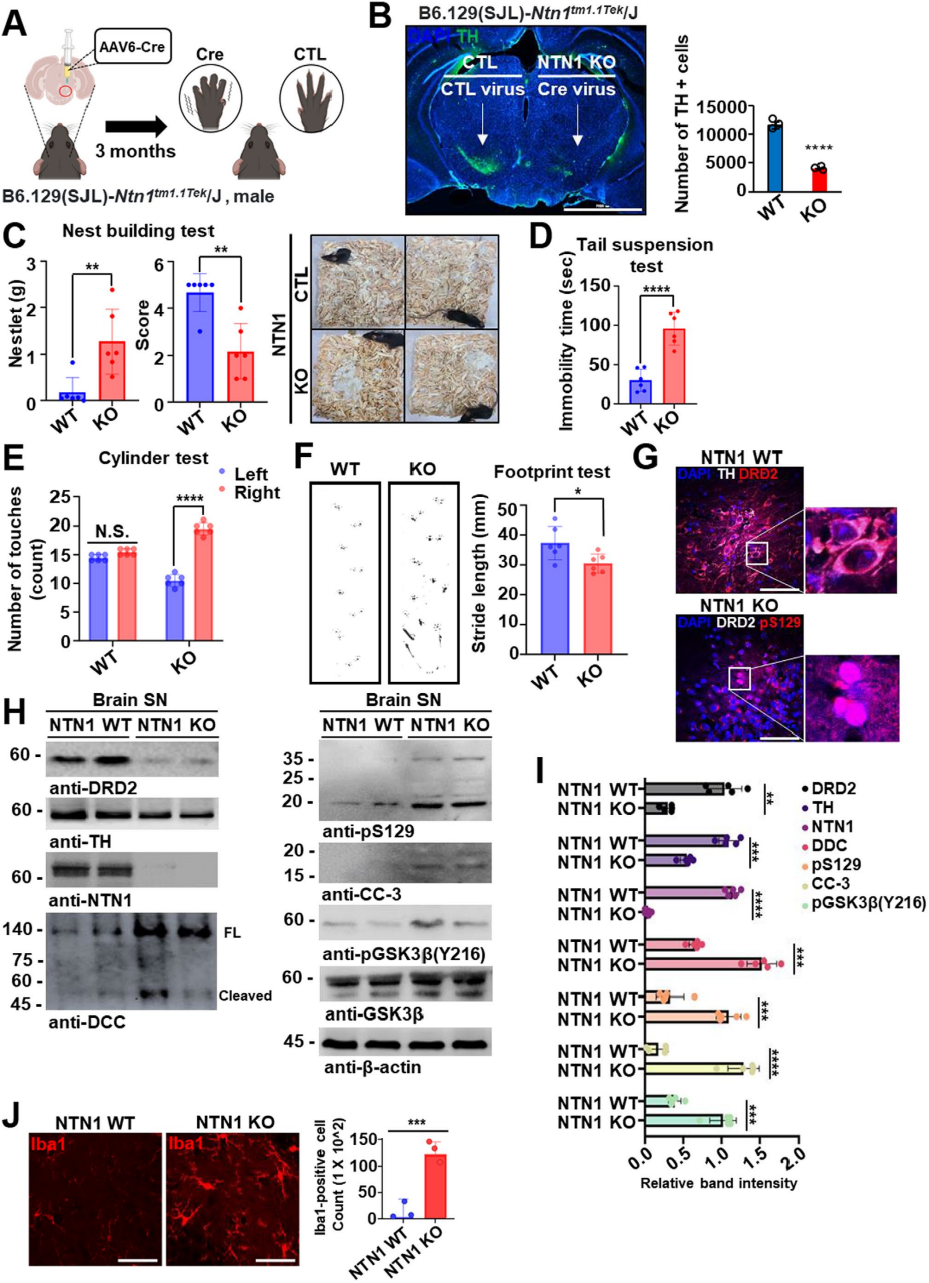
Netrin‐1 knockdown in the SN induces TH loss and motor deficits. (A) Schematic diagram illustrating generation of a Netrin‐1 conditional knockout (KO) mouse model using B6.129(SJL)‐ Ntn1tm1.1Tek/J mice injected with control AAV6 (CTL) or Cre‐expressing AAV6 virus. (B) Representative immunofluorescence images of tyrosine hydroxylase (TH, green) and DAPI (blue) staining in the substantia nigra (SN) of wild‐type (WT) and Netrin‐1 conditional knockout (KO) mice at 8 weeks post‐injection. Scale bar: 2000 μm. Th‐positive cell count in the SN. Individual values and mean ± SEM. *N* = 3 each group; ****p < 0.0001. (C–F) Behavioral assessments (Nest building test (C), Tail suspension test (D), Cylinder test (E), and Footprint test (F)). *N* = 6 animal each group. Bars and error bars represent the mean ± SEM. Statistical significance was determined using a one‐way ANOVA followed by post hoc bonferroni test for multiple group comparison. *p < 0.01; **p < 0.01; ***p < 0.001; ****p < 0.0001; N.S., not significant. (G) Co‐immunofluorescence staining of DRD2 (red)/TH (white, upper panel) or pS129 (red)/DRD2 (white, lower panel) in the SN of Netrin‐1 WT VS Netrin‐1 KO mice (Scale bar: 50 μm). (H) Immunoblot analysis of SN lysates from Netrin‐1 WT and KO mouse brain tissues, assessing the expression levels of DRD2, TH, Netrin‐1, DCC (full‐length and cleaved), pS129 α‐synuclein, cleaved caspase‐3 (CC3), pGSK3β (Y216), total GSK3β, and β‐Actin. Data are representative of three biological replicates. (I) Band densitometer bar graph. Bars and error bars represent the mean ± SEM. Statistical significance was determined using a one‐way ANOVA followed by post hoc Tukey test for multiple group comparison. NTN1 WT *N* = 5; NTN1 KO *N* = 5 each group; **p < 0.01; ***p < 0.001; ****p < 0.0001; N.S., not significant. (J) Immunofluorescence staining of Iba1 to verify neuroinflammation in mouse brain tissue (upper). Number of Iba1‐positive cells quantification bar graph (lower). Data are presented as mean ± SEM (*N* = 3 sections per group from Netrin‐1 WT and KO mice). ***p < 0.001. Scale bar: 50 μm.

The prominent expression of Netrin‐1 in the substantia nigra compacta (SNpc) implies a potential role in maintaining dopaminergic neuronal function in the adult brain, possibly through an autocrine regulatory mechanism. To examine this, we used a loss‐of‐function approach Cre knocking out *netrin‐1* specifically in the adult SN. Netrin‐1 depletion was achieved by injecting stereotaxically and unilaterally AAV6‐Cre virus vector (Cre) into the SN of 3‐month‐old B6.129(SJL)‐Ntn1^tm1.1Tek^/J mice (Figure 4A). The levels of Netrin‐1 and TH in the SN and mice motor behavior were monitored 8 weeks after *netrin‐1* conditional knockout (KO) in the SN. We observed massive TH‐positive cell loss in the brain region affected by AAV6‐Cre virus injection (Figure 4B). To assess whether behavioral deficits commonly observed in Parkinson's disease (PD) models are also present in our system, we performed the Nest building test, Cylinder test, and Footprint test. Mice with Netrin‐1 depletion exhibited significant impairments in all three behavioral paradigms (Figure 4C–F). These findings indicate that the presence of Netrin‐1 in the substantia nigra is critical for maintaining dopaminergic neuronal function and survival. Co‐immunofluorescence staining of the mouse brain substantia nigra revealed a marked reduction in both tyrosine hydroxylase (TH) and dopamine D2 receptor (DRD2) expression, further supporting dopaminergic neuronal loss in Netrin‐1‐deficient mice (Figure 4G). Moreover, we examined the expression levels of DRD2, TH, Netrin‐1 (NTN1), DCC, phosphorylated α‐synuclein (pS129), cleaved caspase‐3 (CC‐3), and phosphorylated GSK3β (Y216) in substantia nigra (SN) tissue lysates from Netrin‐1 wild‐type (WT) and conditional knockout (KO) mice (Figure 4H,I). These molecular alterations were accompanied by enhanced neuroinflammatory responses, as demonstrated by elevated Iba1 immunoreactivity in the substantia nigra (Figure 4J).

### 3.5 Reduced Expression of Netrin‐1 and DRD2 During Aging in hSNCA‐Overexpressing Mice

**FIGURE 5:**
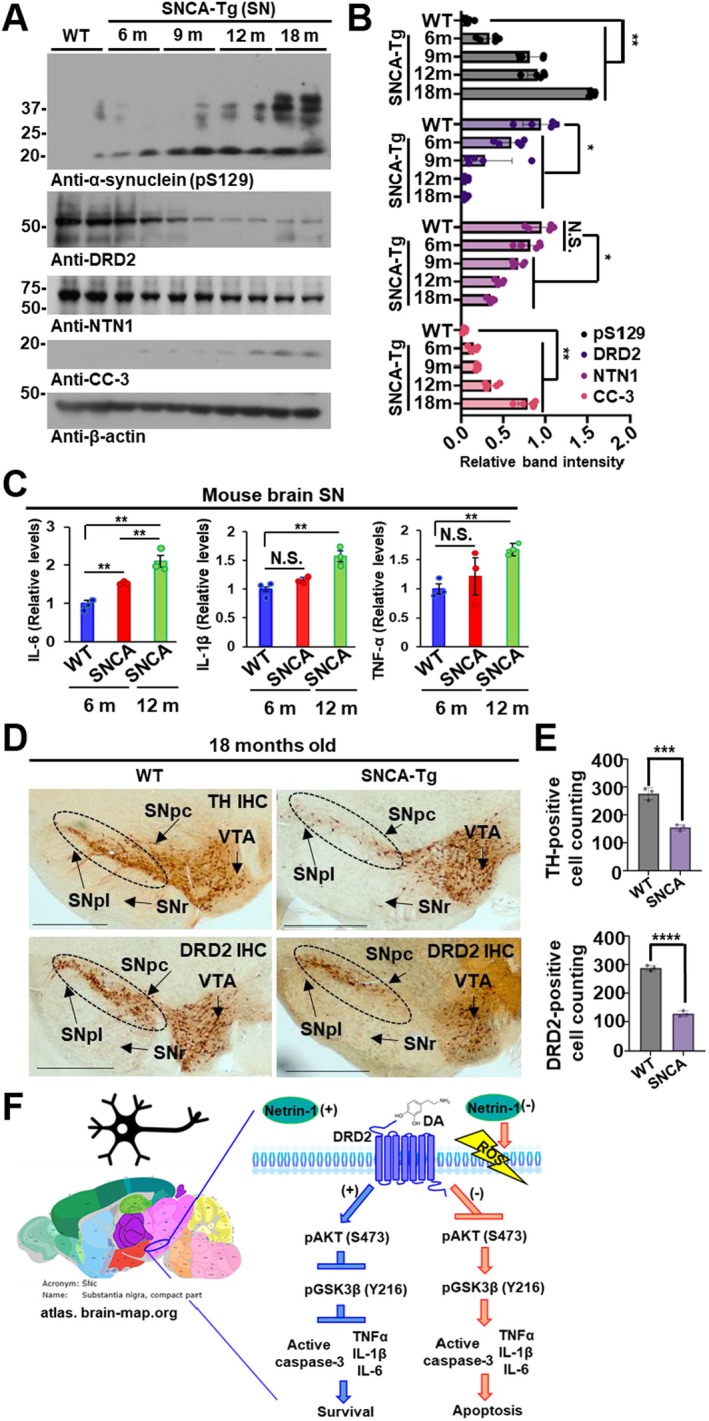
Netrin‐1 prevents age‐dependent progression of neuroinflammation and pathology in hSNCA Tg mice. (A) Immunoblots of pS129, DRD2, Netrin‐1, cleaved caspase‐3 (CC‐3), and β‐Actin in the SN of 18‐month‐old wild‐type (WT) mice and their expression levels in the brain SN region of 6‐, 9‐, 12‐, and 18‐month‐old hSNCA Tg mice. (B) Immunoblot band densitometer bar graph. Bars and error bars represent the mean ± SEM. Statistical significance was determined using a one‐way ANOVA followed by post hoc Tukey test for multiple group comparison. *N* = 5 each group; *p < 0.05; **p < 0.01; N.S., not significant. (C) Proinflammatory cytokines of IL‐6, IL‐1β, and TNF‐α levels in age‐matched WT (*N* = 3) vs. hSNCA Tg mice brain SN lysates (*N* = 3). Bars and error bars represent the mean ± SEM. Statistical significance was determined using a one‐way ANOVA followed by post hoc Bonferroni test for multiple group comparison. **p < 0.01; N.S., not significant. (D) Immunohistochemical staining analysis of TH (upper panel) and DRD2 (lower panel) in the SN brain region of age‐matched WT vs. hSNCA Tg. Scale bar, 1000 μm. (E) Quantification analysis of TH‐ and DRD2‐positive cells. Data represent mean ± SEM. Statistical significance was determined by one‐way ANOVA with Tukey's post hoc test. ***p < 0.001, ****p < 0.0001. (F) A schematic model illustrating how Netrin‐1 supports dopaminergic neuron survival via modulation of DRD2‐mediated signaling in the present study.

We investigated the age‐dependent alterations of key molecules in a Parkinson's disease (PD) context using a transgenic mouse model overexpressing the human α‐synuclein gene (hSNCA), which is critically implicated in PD pathology. Performed immunoblot assays revealed a progressive decline in the expression levels of Netrin‐1 and dopamine D2 receptor (DRD2) in the substantia nigra region of the brain during the aging process; conversely, activated caspase‐3 in hSNCA transgenic (Tg) mice (Figure 5A,B). In parallel, ELISA assays demonstrated a significant increase in the proinflammatory cytokine interleukin‐6 (IL‐6), indicating an age‐related exacerbation of neuroinflammatory responses (Figure 5C). Next, we validated the dopaminergic neuronal changes by performing immunohistochemical staining using antibodies TH and DRD2. This analysis confirmed a marked reduction in DRD2 expression within TH‐positive dopaminergic neurons of the substantia nigra in hSNCA Tg mice only, thereby reinforcing the association between α‐synuclein overexpression, DRD2 downregulation, and PD‐related dopaminergic dysfunction (Figure 5D,E).

## 4 Discussion

In this study, we demonstrate that Netrin‐1 plays a crucial neuroprotective role in Parkinson's disease (PD) by regulating dopaminergic neuronal survival through modulation of DRD2/GSK3β signaling and suppression of oxidative and inflammatory stress. Our findings highlight that Netrin‐1 deficiency contributes to hallmark features of PD pathogenesis, including α‐synuclein hyperphosphorylation, caspase‐3 activation, increased reactive oxygen species (ROS), and neuroinflammation.

Using both cellular and animal models, we showed that recombinant Netrin‐1 protein (rNetrin‐1) significantly reduces ROS generation and α‐synuclein phosphorylation in SH‐SY5Y cells treated with rotenone, a rotenone neurotoxin widely used to mimic PD pathology. Importantly, rNetrin‐1 treatment also suppressed the expression of key proinflammatory cytokines such as IL‐6, TNF‐α, and IL‐1β, suggesting its potent anti‐inflammatory effects in dopaminergic neurons. These results align with previous reports indicating that Netrin‐1 negatively regulates the transcription factor C/EBPβ, a key mediator of neuroinflammatory gene expression [26, 35]. Mechanistically, our data demonstrate that Netrin‐1 modulates dopaminergic signaling through activation of DRD2 and subsequent inhibition of GSK3β activity. We observed that rNetrin‐1 treatment increased phosphorylation of AKT at Ser473 while suppressing hyperphosphorylation of GSK3β at Tyr216, a modification known to enhance its kinase activity and promote neurodegeneration. Given that GSK3β is involved in multiple pro‐apoptotic and pro‐inflammatory pathways [36, 37], its inhibition by Netrin‐1 may serve as a key protective mechanism against PD progression.

In addition, analysis of Netrin‐1 conditional knockout (KO) mice further supported the critical role of endogenous Netrin‐1 in maintaining dopaminergic neuronal integrity. Netrin‐1 depletion in the substantia nigra resulted in severe loss of TH‐ and DRD2‐positive neurons, accompanied by behavioral deficits and elevated expression of apoptotic and inflammatory markers. These observations strongly suggest that endogenous Netrin‐1 is required for dopaminergic neuron survival and functional preservation in the adult brain.

Furthermore, in hSNCA transgenic mice, which overexpress human α‐synuclein, we identified a progressive decline in Netrin‐1 and DRD2 expression in the substantia nigra during aging, correlating with increased caspase‐3 activation and IL‐6 upregulation in the brain SN region. This age‐dependent molecular deterioration reinforces the idea that Netrin‐1 deficiency is a contributing factor to α‐synuclein‐induced dopaminergic degeneration.

Our results also support the regional specificity of DRD2 and DRD1 expression in the dopaminergic system. We showed that DRD2 is predominantly expressed in the substantia nigra, where the soma of dopaminergic neurons resides, whereas DRD1 is mainly found in the striatum, the primary projection site of these neurons [11]. This differential distribution underscores the importance of DRD2 signaling in the cell‐autonomous survival of midbrain dopaminergic neurons. Collectively, our findings propose a model in which Netrin‐1 preserves dopaminergic neuron integrity by activating DRD2‐mediated survival signaling, inhibiting GSK3β activity, and attenuating oxidative and inflammatory insults. These data not only deepen our understanding of Netrin‐1 as a key modulator in PD pathology but also suggest its potential as a therapeutic protein for the treatment or prevention of dopaminergic neurodegeneration. The Netrin‐1–DRD2–GSK3β pathway, this signaling axis may act upstream to modulate several of these mechanisms for instance, by regulating mitochondrial stability, suppressing NF‐κB‐dependent inflammation, and limiting oxidative stress. Thus, our findings complement, rather than exclude, other established pathogenic pathways and help position Netrin‐1 as a critical integrator of dopaminergic survival signaling within the broader PD network.

## Author Contributions

E.H.A. developed the rationale and designed the experiments. Analyzed the data, and wrote the manuscript. E.H.A., L.Y.K. performed most of the experiments and data analysis. E.J.K., S.H.J., Y.H., M.L., D.K.H. and S.S.K. assisted with data analysis, and critically read the manuscript. All authors contributed to the article and approved the submitted version.

## Ethics Statement

Animal care and handling was performed according to NIH animal care guidelines and Emory Medical School guidelines. The protocol was reviewed and approved by the Emory Institutional Animal Care and Use Committee.

## Consent

The authors have nothing to report.

## Conflicts of Interest

The authors declare no conflicts of interest.

## Supporting information


**Figure S1:** Expression levels of dopamine receptor D subtypes in the human substantia nigra. (A) Heatmaps showing DRD1–DRD5 mRNA expression in the SN from healthy human brains. Data were obtained from the Allen Human Brain Atlas. Probe IDs are indicated on the right side. (B) Quantification bar graph of DRD1, DRD3, DRD4 and DRD5 mRNA expression levels in the SN region across multiple donor samples.
**Figure S2:** Regional distribution of DRD2 and DRD1 in the mouse brain. (A) Representative immunofluorescence images showing DRD2 expression (green) in the striatum (top), hippocampus (middle), and substantia nigra (bottom) of adult mouse brain sections. Nuclei were counterstained with DAPI (blue). (B) Representative immunofluorescence images showing DRD1 expression (green) in the same regions. Right panels show magnified views of boxed areas. Scale bar: 1 mm.
**Table S1:** Information of Allen human brain dataset.
Normalized z‐scores for each gene and probe in substantia nigra samples from adult healthy donors, obtained from the Allen Human Brain Atlas. Donor demographics and probe details are included.
**Table S2:** Information on antibodies used in the present study.
**Table S3:** Information on experimental analysis kits used in the present study.

## Data Availability

The authors declare that all data supporting the findings of this study is available within the article and its Supporting Information files or from the corresponding author and 1st author upon request via email.
