# Supplementary material for: Netrin‐1 Inhibits Neuroinflammation by Modulating DRD2/GSK3β Signaling and Suppressing ROS in a Parkinson's Disease Model
Source: CNS Neurosci Ther. 2025 Nov 17;31(11):e70651. doi: 10.1111/cns.70651 (PMC12623146; doi:10.1111/cns.70651)
Supplement: Supplementary file 1 — Figure S1: Expression levels of dopamine receptor D subtypes in the human substantia nigra. (A) Heatmaps showing DRD1–DRD5 mRNA expression in the SN from healthy human brains. Data were obtained from the Allen Human Brain Atlas. Probe IDs are indicated on the right side. (B) Quantification bar graph of DRD1, DRD3, DRD4 and DRD5 mRNA expression levels in the SN region across multiple donor samples. Figure S2: Regional distribution of DRD2 and DRD1 in the mouse brain. (A) Representative immunofluorescence images showing DRD2 expression (green) in the striatum (top), hippocampus (middle), and substantia nigra (bottom) of adult mouse brain sections. Nuclei were counterstained with DAPI (blue). (B) Representative immunofluorescence images showing DRD1 expression (green) in the same regions. Right panels show magnified views of boxed areas. Scale bar: 1 mm. Table S1: Information of Allen human brain dataset. Normalized z‐scores for each gene and probe in substantia nigra samples from adult healthy donors, obtained from the Allen Human Brain Atlas. Donor demographics and probe details are included. Table S2: Information on antibodies used in the present study. Table S3: Information on experimental analysis kits used in the present study. [file CNS-31-e70651-s001.pdf]

Supplementary Figure 1

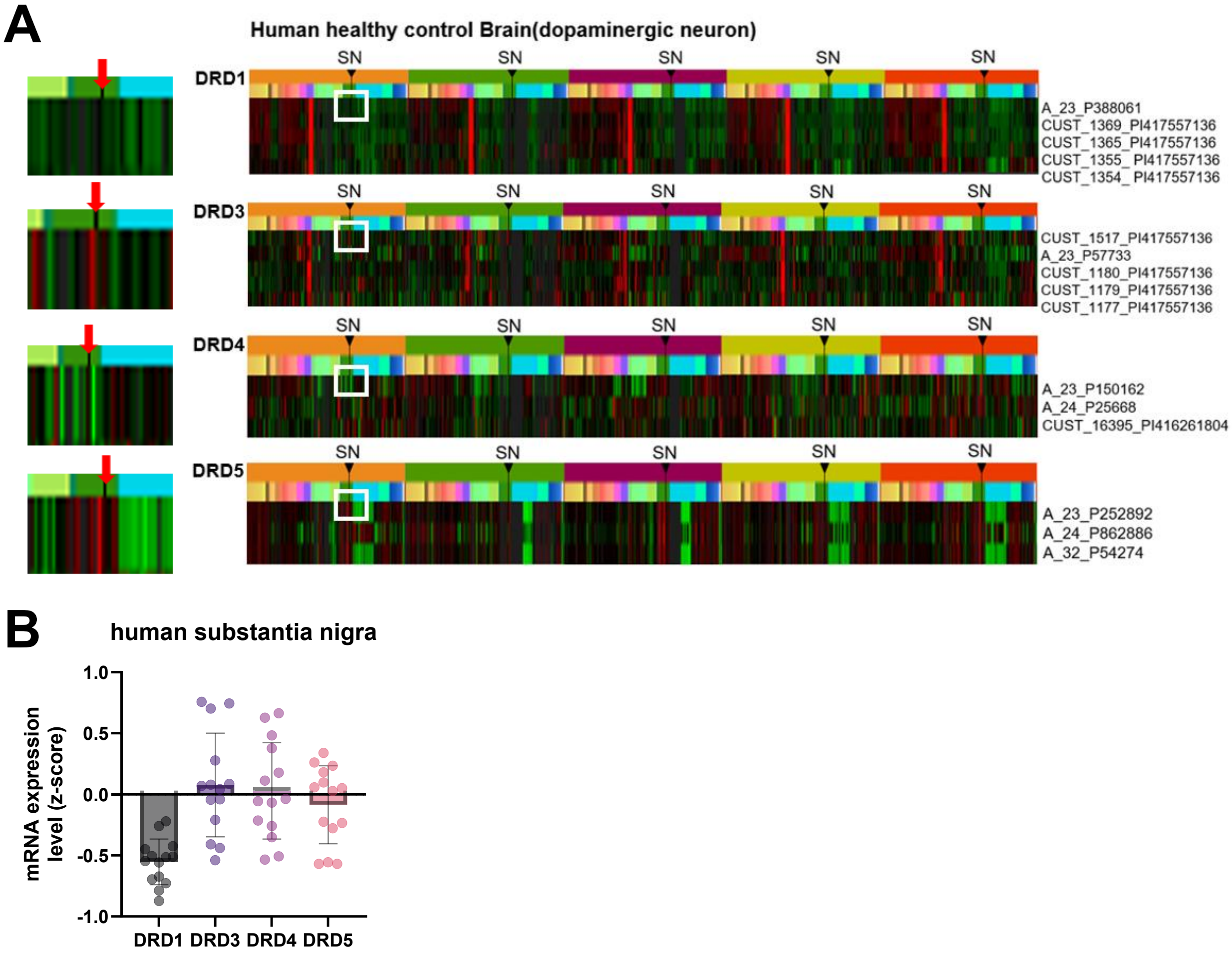

Supplementary Figure 2

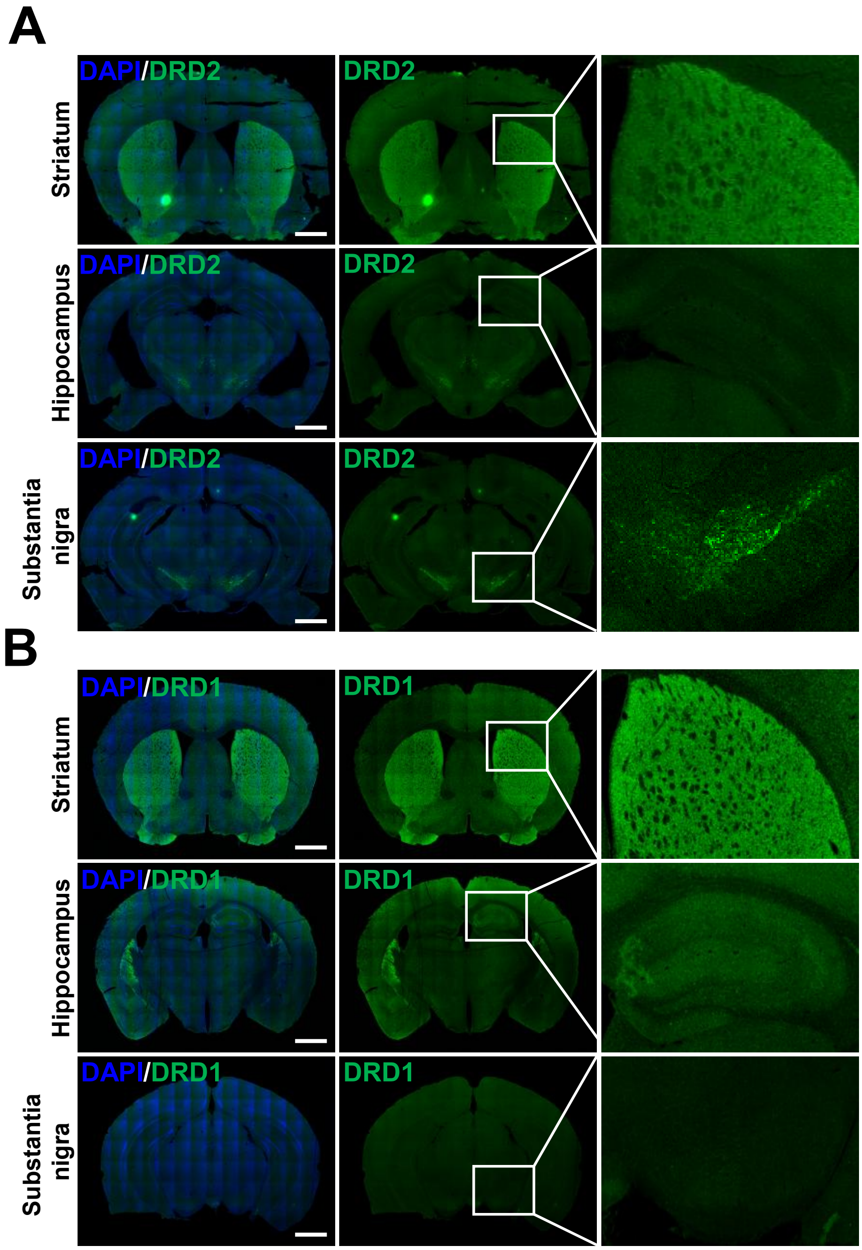

Supplementary Table 1

Gene name	Probe name	Structure	Donor	z-score
DRD1	A_23_P388061	Substantia nigra(SN)	H0351.2001, 24 yrs, M, Black or African American	-0.726725
	A_23_P388061	Substantia nigra(SN)	H0351.2002, 39 yrs, M, Black or African American	-0.44977
	A_23_P388061	Substantia nigra(SN)	H0351.1009, 57 yrs, M, White or Caucasian	-0.514394
	A_23_P388061	Substantia nigra(SN)	H0351.1015, 49 yrs, F, Hispanic	-0.672785
	CUST_1369_PI417557136	Substantia nigra(SN)	H0351.2001, 24 yrs, M, Black or African American	-0.510707
	CUST_1369_PI417557136	Substantia nigra(SN)	H0351.2002, 39 yrs, M, Black or African American	-0.220372
	CUST_1369_PI417557136	Substantia nigra(SN)	H0351.1009, 57 yrs, M, White or Caucasian	-0.258457
	CUST_1369_PI417557136	Substantia nigra(SN)	H0351.1012, 31 yrs, M, White or Caucasian	-0.50602
	CUST_1369_PI417557136	Substantia nigra(SN)	H0351.1015, 49 yrs, F, Hispanic	-0.545865
	CUST_1365_PI417557136	Substantia nigra(SN)	H0351.2001, 24 yrs, M, Black or African American	-0.78717
	CUST_1365_PI417557136	Substantia nigra(SN)	H0351.2002, 39 yrs, M, Black or African American	-0.558163
	CUST_1365_PI417557136	Substantia nigra(SN)	H0351.1009, 57 yrs, M, White or Caucasian	-0.424671
	CUST_1365_PI417557136	Substantia nigra(SN)	H0351.1012, 31 yrs, M, White or Caucasian	-0.696028
	CUST_1365_PI417557136	Substantia nigra(SN)	H0351.1015, 49 yrs, F, Hispanic	-0.873037
DRD2	A_24_P283834	Substantia nigra(SN)	H0351.2001, 24 yrs, M, Black or African American	1.60929
	A_24_P283834	Substantia nigra(SN)	H0351.2002, 39 yrs, M, Black or African American	1.50154
	A_24_P283834	Substantia nigra(SN)	H0351.1009, 57 yrs, M, White or Caucasian	1.64475
	A_24_P283834	Substantia nigra(SN)	H0351.1012, 31 yrs, M, White or Caucasian	1.72107
	A_24_P283834	Substantia nigra(SN)	H0351.1015, 49 yrs, F, Hispanic	2.4759
	A_24_P283834	Substantia nigra(SN)	H0351.1016, 55 yrs, M, White or Caucasian	1.09437
	CUST_1494_PI417557136	Substantia nigra(SN)	H0351.2001, 24 yrs, M, Black or African American	2.59744
	CUST_1494_PI417557136	Substantia nigra(SN)	H0351.2002, 39 yrs, M, Black or African American	2.75259
	CUST_1494_PI417557136	Substantia nigra(SN)	H0351.1009, 57 yrs, M, White or Caucasian	2.7435
	CUST_1494_PI417557136	Substantia nigra(SN)	H0351.1012, 31 yrs, M, White or Caucasian	2.76961
	CUST_1494_PI417557136	Substantia nigra(SN)	H0351.1015, 49 yrs, F, Hispanic	2.49636
	CUST_1494_PI417557136	Substantia nigra(SN)	H0351.1016, 55 yrs, M, White or Caucasian	0.760723
DRD3	A_23_P57733	Substantia nigra(SN)	H0351.1012, 31 yrs, M, White or Caucasian	0.702142
	A_23_P57733	Substantia nigra(SN)	H0351.2001, 24 yrs, M, Black or African American	0.757796
	A_23_P57733	Substantia nigra(SN)	H0351.1009, 57 yrs, M, White or Caucasian	-0.539402
	A_23_P57733	Substantia nigra(SN)	H0351.1015, 49 yrs, F, Hispanic	-0.0410032
	A_23_P57733	Substantia nigra(SN)	H0351.1016, 55 yrs, M, White or Caucasian	-0.209812
	CUST_1517_PI417557136	Substantia nigra(SN)	H0351.2001, 24 yrs, M, Black or African American	-0.438832
	CUST_1517_PI417557136	Substantia nigra(SN)	H0351.1009, 57 yrs, M, White or Caucasian	0.743719
	CUST_1517_PI417557136	Substantia nigra(SN)	H0351.1015, 49 yrs, F, Hispanic	0.0863364
	CUST_1517_PI417557136	Substantia nigra(SN)	H0351.1012, 31 yrs, M, White or Caucasian	0.0698534
	CUST_1179_PI417557136	Substantia nigra(SN)	H0351.2001, 24 yrs, M, Black or African American	0.277019
	CUST_1179_PI417557136	Substantia nigra(SN)	H0351.1009, 57 yrs, M, White or Caucasian	-0.0446174
	CUST_1179_PI417557136	Substantia nigra(SN)	H0351.1012, 31 yrs, M, White or Caucasian	-0.404859
	CUST_1179_PI417557136	Substantia nigra(SN)	H0351.1015, 49 yrs, F, Hispanic	0.0796793
	CUST_1179_PI417557136	Substantia nigra(SN)	H0351.1016, 55 yrs, M, White or Caucasian	0.0421151
DRD4	A_23_P150162	Substantia nigra(SN)	H0351.2001, 24 yrs, M, Black or African American	-0.5068
	A_23_P150162	Substantia nigra(SN)	H0351.1009, 57 yrs, M, White or Caucasian	-0.258746
	A_23_P150162	Substantia nigra(SN)	H0351.1012, 31 yrs, M, White or Caucasian	0.664333
	A_23_P150162	Substantia nigra(SN)	H0351.1015, 49 yrs, F, Hispanic	0.627665
	A_23_P150162	Substantia nigra(SN)	H0351.1016, 55 yrs, M, White or Caucasian	-0.534906
	CUST_16395_PI416261804	Substantia nigra(SN)	H0351.1016, 55 yrs, M, White or Caucasian	0.178089
	CUST_16395_PI416261804	Substantia nigra(SN)	H0351.2001, 24 yrs, M, Black or African American	0.377865
	CUST_16395_PI416261804	Substantia nigra(SN)	H0351.1009, 57 yrs, M, White or Caucasian	0.1126
	CUST_16395_PI416261804	Substantia nigra(SN)	H0351.1012, 31 yrs, M, White or Caucasian	-0.351493
	CUST_16395_PI416261804	Substantia nigra(SN)	H0351.1015, 49 yrs, F, Hispanic	-0.0379713
	A_24_P25668	Substantia nigra(SN)	H0351.2001, 24 yrs, M, Black or African American	0.481735
	A_24_P25668	Substantia nigra(SN)	H0351.1009, 57 yrs, M, White or Caucasian	-0.214069
	A_24_P25668	Substantia nigra(SN)	H0351.1012, 31 yrs, M, White or Caucasian	-0.0560399
	A_24_P25668	Substantia nigra(SN)	H0351.1016, 55 yrs, M, White or Caucasian	-0.0679435
DRD5	A_23_P252892	Substantia nigra(SN)	H0351.2001, 24 yrs, M, Black or African American	0.339326
	A_23_P252892	Substantia nigra(SN)	H0351.1009, 57 yrs, M, White or Caucasian	-0.224604
	A_23_P252892	Substantia nigra(SN)	H0351.1012, 31 yrs, M, White or Caucasian	0.233652
	A_23_P252892	Substantia nigra(SN)	H0351.1015, 49 yrs, F, Hispanic	0.098049
	A_23_P252892	Substantia nigra(SN)	H0351.1016, 55 yrs, M, White or Caucasian	0.0501022
	A_24_P862886	Substantia nigra(SN)	H0351.2001, 24 yrs, M, Black or African American	-0.555492
	A_24_P862886	Substantia nigra(SN)	H0351.1012, 31 yrs, M, White or Caucasian	-0.23378
	A_24_P862886	Substantia nigra(SN)	H0351.1015, 49 yrs, F, Hispanic	-0.27686
	A_24_P862886	Substantia nigra(SN)	H0351.1016, 55 yrs, M, White or Caucasian	-0.569613
	A_32_P54274	Substantia nigra(SN)	H0351.2001, 24 yrs, M, Black or African American	0.181271
	A_32_P54274	Substantia nigra(SN)	H0351.1009, 57 yrs, M, White or Caucasian	-0.569236
	A_32_P54274	Substantia nigra(SN)	H0351.1012, 31 yrs, M, White or Caucasian	0.26008
	A_32_P54274	Substantia nigra(SN)	H0351.1015, 49 yrs, F, Hispanic	0.0272932
	A_32_P54274	Substantia nigra(SN)	H0351.1016, 55 yrs, M, White or Caucasian	0.0569648
SNCA	A_23_P29939	Substantia nigra(SN)	H0351.2001, 24 yrs, M, Black or African American	0.918524
	A_23_P29939	Substantia nigra(SN)	H0351.2002, 39 yrs, M, Black or African American	0.909573
	A_23_P29939	Substantia nigra(SN)	H0351.1009, 57 yrs, M, White or Caucasian	0.69586
	A_23_P29939	Substantia nigra(SN)	H0351.1012, 31 yrs, M, White or Caucasian	0.843342
	A_23_P29939	Substantia nigra(SN)	H0351.1015, 49 yrs, F, Hispanic	1.24621
	A_23_P29939	Substantia nigra(SN)	H0351.1016, 55 yrs, M, White or Caucasian	0.99691
	A_32_P109653	Substantia nigra(SN)	H0351.2001, 24 yrs, M, Black or African American	0.557793
	A_32_P109653	Substantia nigra(SN)	H0351.2002, 39 yrs, M, Black or African American	0.550617
	A_32_P109653	Substantia nigra(SN)	H0351.1009, 57 yrs, M, White or Caucasian	0.890565
	A_32_P109653	Substantia nigra(SN)	H0351.1012, 31 yrs, M, White or Caucasian	0.667298
	A_32_P109653	Substantia nigra(SN)	H0351.1015, 49 yrs, F, Hispanic	0.924157
	A_32_P109653	Substantia nigra(SN)	H0351.1016, 55 yrs, M, White or Caucasian	0.629845

Supplementary Table 2

- **Antibodies information**

Antibody name	Company	Dilution	Catalog number
DRD2	LS Bio	1:250	LS-C159490, Mouse
DRD2	Proteintech	1:500	55084-1-AP, Rabbit
DRD2	ThermoFisher	1:500	#75-230, Mouse
DCC	Santa Cruz	1:500	SC-515834, Mouse
DRD1	Invitrogen	1:500	#702593, Rabbit
α-synuclein pS129	LS Bio	1:250	LS-C380861-1, Rabbit
TH	Cell Signaling	1:1000	#2792, Rabbit
TH	R&D Systems	1:1000	MAB7566, Mouse
CC-3	Cell Signaling	1:100	#9661, Rabbit
β-actin	Cell Signaling	1:1000	#4967, Rabbit
Iba1	Invitrogen	1:1000	MA5-27726, Mouse
NTN1	Abcam	1:500	ab126729, Rabbit
NTN1	Abcam	1:500	ab122903, Goat
GSK3β	Cell Signaling	1:1000	#9315S, Rabbit
GSK3β (Tyr216)	Proteintech	1:500	29125-1-AP,Rabbit
Pan-AKT	Abcam	1:1000	ab8805, Rabbit
AKT(Ser 473)	Abcam	1:1000	ab81283, Rabbit

Supplementary Table 3

- **Material information**

ELISA Kit	Company	Catalog number
IL-6	Invitrogen	#EH2IL6, Human
	Invitrogen	#88-7064-88, Mouse
IL-1β	Invitrogen	#BMS224-2, Human
	Invitrogen	#88-7013A-76, Mouse
TNF-α	Invitrogen	#BMS223-4, Human
	Invitrogen	#BMS607-3, Mouse
DCFDA	Abcam	ab113851
TUNEL (Red)	Abcam	ab66110
